# Alzheimer’s Disease: A Major Challenge in the Effective Treatment of Depression in the Elderly

**Published:** 2008-06-15

**Authors:** Shailendra Kapoor

## To the Editor:

The recent article by Snowden et al (1) is illuminating but does not mention the close relationship between late-life depression and Alzheimer's disease. Late-life depression may actually be an early manifestation of Alzheimer's disease itself. Failure to recognize subtle signs and symptoms can delay appropriate treatment, which is a major challenge in effectively treating depression in the elderly.

Rates of depression as high as 51% have been reported in patients with Alzheimer's disease (2). In fact, recent studies indicate that late-life depression may be a substantial risk factor for Alzheimer's disease (3). Wetherell et al have reported that elderly patients with depression are almost 4 times more likely than those without depression to develop Alzheimer's disease, and this risk is even greater in those patients who report symptoms such as decreased energy levels or a decline in interest (4). Patients with Alzheimer's disease who develop depression show decreased frontal lobe activity and glucose hypometabolism, especially in the bilateral superior gyri and the left anterior cingulated gyri (5).

Physicians should be aware of the close relationship between Alzheimer's disease and depression. Recognizing Alzheimer's disease and appropriately managing it can go a long way toward effectively treating late-life depression.

## References

[1] Snowden M, Steinman L, Frederick J (2008). Treating depression in older adults: challenges to implementing the recommendations of an expert panel. Prev Chronic Dis.

[2] Migliorelli R, Teson A, Sabe L, Petracchi M, Leiguarda R, Starkstein SE (1995). Prevalence and correlates of dysthymia and major depression among patients with Alzheimer's disease. Am J Psychiatry.

[3] Green RC, Cupples LA, Kurz A, Auerbach S, Go R, Sadovnick D (2003). Depression as a risk factor for Alzheimer disease: the MIRAGE Study. Arch Neurol.

[4] Wetherell JL, Gatz M, Johansson B, Pedersen NL (1999). History of depression and other psychiatric illness as risk factors for Alzheimer disease in a twin sample. Alzheimer Dis Assoc Disord.

[5] Hirono N, Mori E, Ishii K, Ikejiri Y, Imamura T, Shimomura T (1998). Frontal lobe hypometabolism and depression in Alzheimer's disease. Neurology.

